# No Row

**Published:** 1897-02

**Authors:** 


No Row.—In the December Journal, in speaking of Dr. Rosser's resignation as superintendent of the Insane Asylum at Terrell, we asked "What's the row now?"

Friends of Dr. Rosser have written us that this is an unmerited reflection on the doctor; and we think it is due him to say, that such was not intended, by any means. At the time the paragraph was written, no explanation of his reasons for resigning had reached us,—and as we had understood that the doctor would stand for re-appointment, and that he would make a specialty of insanity and nervous diseases, his resignation, within a few weeks of the expiration of his term of office, struck us as remarkable, at least. And, especially, when taken in connection with the unfortunate fact that some asylum resignations have been resignations only by courtesy—a "row" of more or less magnitude figuring in the background. In light of facts which have since come to our knowledge, we see how a suspicion, suggested, as was done in the paragraph referred to—however justified at the time and under the circumstances—was an injustice to Dr. Rosser, and it gives us pleasure to now state that there was no row. His resignation was entirely voluntary, though it had not been contemplated, we believe.

Dr. Rosser had, prior to his appointment, been associated in practice with Dr. Letcher, of Dallas; and had certain other interests in common with him. Dr. Letcher's death, in November, made it advisable for Dr. Rosser's best interests, to return to Dallas, where he expects to take up the practice, in part at least, of the late firm, and look after other matter considered of more importance, we learn, than the salary of superintendent of the asylum.

There were other reasons, also, but this will be sufficient. No complaint of his administration ever reached us. On the contrary, he is credited with a clean, efficient and economical administration, and it is pointed out that he reduced the yearly expenditures something like forty thousand dollars.

Dr. Rosser is one of the foremost of the younger generation of Texas physicians; a staunch friend of the JOURNAL, and far be it from us to intentionally hit him a lick; we reserve the licks for our enemies. whom we *love to hate.*

